# Persistent elevation of urine aquaporin-2 during water loading in a child with nephrogenic syndrome of inappropriate antidiuresis (NSIAD) caused by a R137L mutation in the V2 vasopressin receptor

**DOI:** 10.1186/1687-9856-2012-3

**Published:** 2012-02-10

**Authors:** Clement C Cheung, Melissa A Cadnapaphornchai, Sayali A Ranadive, Stephen E Gitelman, Stephen M Rosenthal

**Affiliations:** 1 Department of Pediatrics, Division of Endocrinology, University of California, San Francisco, San Francisco, CA 94143; 2 Department of Pediatrics, Division of Nephrology, University of Colorado Denver, Aurora, CO 80045; 3 Pediatric Endocrinology, Palo Alto Medical Foundation, Fremont, CA 94538

**Keywords:** Aquaporin-2, NSIAD, V2R, Water Loading

## Abstract

Nephrogenic Syndrome of Inappropriate Antidiuresis (NSIAD) is a novel disease caused by a gain-of-function mutation in the V2 vasopressin receptor (V2R), which results in water overload and hyponatremia. We report the effect of water loading in a 3-year old boy with NSIAD, diagnosed in infancy, to assess urine aquaporin-2 (AQP2) excretion as a marker for V2R activation, and to evaluate the progression of the disease since diagnosis. The patient is one of the first known NSIAD patients and the only patient with a R137L mutation. Patient underwent a standard water loading test in which serum and urine sodium and osmolality, serum AVP, and urine AQP2 excretion were measured. The patient was also evaluated for *ad lib *fluid intake before and after the test. This patient demonstrated persistent inability to excrete free water. Only 39% of the water load (20 ml/kg) was excreted during a 4-hour period (normal ≥ 80-90%). Concurrently, the patient developed hyponatremia and serum hypoosmolality. Serum AVP levels were detectable at baseline and decreased one hour after water loading; however, urine AQP2 levels were elevated and did not suppress normally during the water load. The patient remained eunatremic but relatively hypodipsic during *ad lib *intake. In conclusion, this is the first demonstration in a patient with NSIAD caused by a R137L mutation in the V2R that urine AQP2 excretion is inappropriately elevated and does not suppress normally with water loading. In addition, this is the first longitudinal report of a pediatric patient with NSIAD diagnosed in infancy who demonstrates the ability to maintain eunatremia during *ad lib *dietary intake.

## Introduction

Normal fluid balance requires an intact thirst mechanism and normal free water excretion by the kidneys, mediated by arginine vasopressin (AVP), also known as antidiuretic hormone (ADH). AVP exerts its antidiuretic action *via *the V2 vasopressin receptor (V2R) in the basolateral membrane of renal collecting duct cells. Binding and activation of the V2R, a G-protein coupled receptor (GPCR), increases intracellular cAMP and mediates shuttling of the water channel aquaporin-2 (AQP2) from cytosolic storage vesicles to the apical membrane of collecting duct cells, resulting in increased water permeability and antidiuresis [1]. A fraction of this AQP2 is excreted in the urine and has been found to be a useful marker of V2R activity [2]. Water loading in a normal individual suppresses plasma AVP levels and attenuates antidiuresis as a result of decreased AQP2 shuttling to the apical membrane of collecting duct cells. Consequently, less AQP2 is shed into the urine [3,4].

We have previously described a novel syndrome of impaired water excretion mediated by gain-of-function mutations in the X-linked gene for V2R in two unrelated male infants who presented with irritability or seizures and hyponatremia [5]. Their clinical and laboratory findings were consistent with the syndrome of inappropriate secretion of antidiuretic hormone (SIADH) yet their AVP levels were undetectable. We have termed this condition "nephrogenic syndrome of inappropriate antidiuresis" (NSIAD). Each patient carries a missense mutation in codon 137 of *AVPR2*, which results in a change from arginine to cysteine (R137C) in one patient and to leucine (R137L) in the other [5]. Codon 137 is part of a highly conserved DRY/H motif located at the junction of the third transmembrane domain and the second intracellular loop of class 1 GPCRs. This motif is critical for G-protein coupling [6] and the two mutations each resulted in a constitutively active V2R [5].

Since our initial description of NSIAD, many reports from around the world, including individual and family studies, have characterized the clinical course of this syndrome [7-12]. All of these patients have the R137C V2R mutation. Here we report the clinical course of the only known patient with NSIAD caused by the more potent R137L V2R mutation [5]. This patient demonstrated the ability to maintain eunatremia during *ad lib *dietary intake. However, urine AQP2 levels were elevated and did not suppress normally during a standard water loading test, consistent with a gain-of-function mutation of V2R.

## Patient and methods

### Patient

CS first presented at 2.5 months of age with seizures and hyponatremia (118-120 mmol/L). His laboratory studies suggested SIADH; however, he had undetectable AVP levels on repeated occasions. DNA sequencing revealed a missense mutation in his *AVPR2 *gene, resulting in a R137L gain-of-function mutation of the V2R [5]. Fluid restriction and oral urea resulted in eunatremia and normal growth and development. The patient was lost to follow-up between age 20-32 months, during which time urea was given only intermittently and ultimately discontinued by the family at age 30 months. Despite inconsistent or complete lack of intake of urea, periodic measurements of serum sodium concentration by his pediatrician were always within the normal range. He was admitted to our institution at age 36 months to assess his water metabolism and to determine need for ongoing urea treatment.

### Water loading test

A water loading test [4,13,14] was performed in the Pediatric Clinical Research Center at our institution. A peripherally-inserted central catheter and a Foley catheter were placed 24 hours prior to the test. The patient was monitored without fluid restriction for 24 hr. Baseline urine and blood were collected at 60 and 30 minutes prior to administration of water [20 mL/kg (360 mL) over 30 minutes via nasogastric tube]. Urine and blood samples were collected every 30 and 60 minutes, respectively, for 4 hours for measurement of sodium and osmolality. In addition, serum AVP (Quest diagnostics) [5] and urine AQP2, creatinine, and urine volume were measured. After the test, the patient was observed on *ad libitum *dietary intake. Intake and output were monitored for an additional 72 hours. Of note, the same reference laboratory for serum AVP measurement was used throughout this child's life.

### Preparation of urine samples for urinary AQP2 analysis

Urine samples were centrifuged at 4,000 g for 5 min to remove any debris. The supernatant was diluted 1:1 with 2 × sodium dodecyl sulfate (SDS) sample buffer containing 2-mercaptoethanol. The samples were heated for 15 min at 65°C and stored at -20°C until analyzed. Just before loading for electrophoresis, samples were warmed to 37°C.

### Development of AQP2 antibody

As described previously [15], a rabbit polyclonal antibody against AQP2 was prepared by Gene-med Biotechnologies Inc., (San Francisco, CA, USA) using a synthetic peptide (VELHSPQSLPRGSKA) from the COOH terminus of AQP2 [16]. The peptide was conjugated to keyhole limpit hemocyanin (KHL) by a cystein sulfhydryl linkage. Test bleedings were screened by ELISA. Final titers were reported to be > 100,000. In western blot analysis, the AQP2 antibody showed no reactivity to BSA.

### Urine AQP2 western blot

AQP2 peptide was conjugated to BSA using the Imject Immunogen EDC conjugation kit (Pierce, Rockford, IL, USA). The AQP2 peptide conjugated BSA (AQP2-BSA) at varying concentrations and 10 μl of the patient's sample at each time point were resolved on a 12% SDS-polyacrylamide gel and transferred to a polyvinylidine difluoride membrane. The membrane was incubated with the rabbit anti-human AQP2 antibody at 4°C overnight, washed, and incubated with anti-rabbit IgG horseradish peroxidase antibody (Amersham, Piscataway, NJ, USA) [15]. Immunoreactive bands were visualized by enhanced chemiluminescence (NEN Life Science). The bands of the AQP2-BSA standards and the nonglycosylated form of AQP2 on the film were scanned and analyzed using NIH image software.

### Calculation of urinary AQP2 excretion

After densitometry measurements, a standard curve was constructed of known amounts of AQP2-BSA versus densitometry measurements, and the densitometry measurements of the urine samples were converted to a numerical value calculated from the curve (Prism, Graph Pad, San Diego, CA, USA). Numerical values were reported as pmol/mg creatinine. In initial studies, concentrations of AQP2 peptide conjugated BSA included 10, 20, 30, and 50 ng [15]. However, with these concentrations of AQP2-BSA, the patient's samples for urine AQP2 were too high to accurately measure. Repeat immunoblots were performed with AQP2-BSA at 50, 100, 150, and 200 ng as described above.

## Results

### Pre-water loading test

As initially reported at the time of NSIAD diagnosis, serum AVP was undetectable with concurrent hyponatremia, serum hypoosmolality, and inappropriately concentrated urine (Table 1). Following initiation of water restriction and urea treatment [17], serum sodium and osmolality normalized, associated with measurable serum AVP of 5.5 pg/mL (Table 1). Despite *ad libitum *fluid intake and subsequent discontinuation of urea, these values remained normal (Table 1).

**Table 1:** Serum sodium (Na) concentration, serum osmolality (Osm), serum AVP, and urine osmolality from initial evaluation at 2.5 months to 3 years of age

Age	Events	Serum Na (134-143 mmol/L)	Serum Osm (285-293 mOsm/kg)	Serum AVP (1.0-13.3 pg/mL)	Urine Osm (300-900 mOsm/kg)
2.5 m	Initial presentation	118	247	< 1	390
5.5 m	After 1 month of water restriction and urea	142	295	5.5	n/a*
3 yr	Off urea for > 6 months; *ad lib *intake	139	295	3.1	628

*n/a = not available

### Water loading test

Prior to the water loading test, the patient's serum sodium concentration was 135 mmol/L, and serum and urine osmolality were 283 mmol/L and 857 mmol/kg, respectively. An hour after the 20 mL/kg water load, serum sodium was 128 mmol/L, and serum and urine osmolality were 272 mmol/L and 709 mmol/kg, respectively (Table 2). Over the next three hours, his serum sodium concentration remained stable and was 127 mmol/L at the conclusion of the test, at which time his serum osmolality had decreased to 267 mmol/L. Urine osmolality was inappropriately increased to 884 mmol/kg, exceeding the baseline value. Of note, serum AVP was 2.6 pg/mL at baseline and decreased to 1.1 pg/mL by one hour after water loading. Urine output during the 4-hour test was 140 mL, which was only 39% of the water load (normal ≥ 80-90% of the water load).

**Table 2:** Serum sodium, serum osmolality, and urine osmolality during standard water loading test (20 mL/kg) at 3 years of age

Water Loading Test			
**Time** **(min)**	**Serum Na** **(134-143 mmol/L)**	**Serum Osm** **(285-294 mOsm/kg)**	**Urine Osm** **(300-900 mOsm/kg)**

-60	135	283	857
+60	128	272	709
+120	128	266	753
+180	128	268	842
+240	127	267	884

### Intake and output during *ad lib *fluid intake

During the subsequent 3 days following the water loading test, the patient had free access to liquid based on his thirst. His daily intake was between 50-55 mL/kg/24 h (normal fluid intake for weight is 75-80 mL/kg/24 h) [18] whereas his 24 hour urine output remained at 30-35 mL/kg/24 hr. His serum sodium concentration was maintained between 131 and 139 mmol/L during this time.

### Urine aquaporin 2

**Figure 1:**
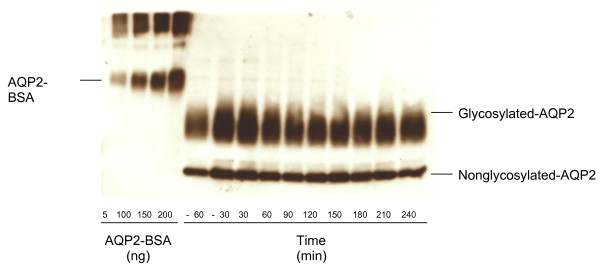
**Western immunoblot showing urine AQP2 excretion from 60 minutes prior to 240 minutes following oral water load**. AQP2-BSA standards included 50, 100, 150, and 200 ng.

**Figure 2:**
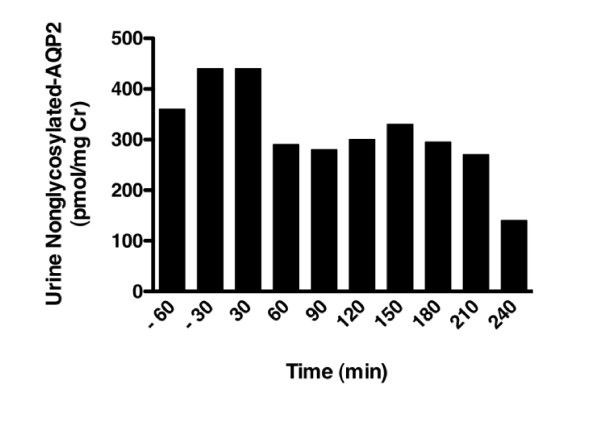
**Quantitative assessment of nonglycosylated AQP2 excretion in pmol/mg creatinine from 60 minutes prior to 240 minutes following oral water load**. Sample values for the patient were corrected for urinary creatinine.

**Figure 3:**
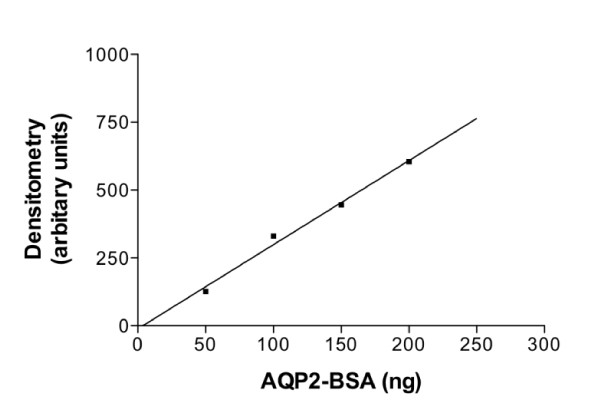
**Standard curve for urine AQP2-BSA measurement**. The y-axis represents densitometry measurement and the x-axis represents the corresponding nanogram value for AQP2-BSA.

Quantitative immunoblot assay for urine AQP2 showed an abnormally elevated baseline of 420 pmol/mg Cr, which did not suppress normally for at least 3 hr after water loading (87-98% of baseline, where normal is 5-17% of baseline) [15] (Figures 1, 2). The AQP2-BSA standard curve for densitometry measurement is shown in Figure 3[5].

## Discussion

This is the first demonstration in a patient with NSIAD caused by the R137L V2R mutation of urinary AQP2 excretion which was markedly elevated at baseline and which did not suppress normally in a standard water loading test. Our patient's baseline urine AQP2 excretion exceeded that seen in normal adults following 12 hours of water deprivation (7-24 pmol/mg creatinine; personal communication, F. Umenishi), a condition known to increase urine AQP2 excretion [3]. These findings of increased urinary AQP2 in our patient are consistent with a gain of function mutation in V2R.

Persistent urinary excretion of AQP2 during water loading has been reported in one patient with NSIAD [12] caused by the less potent R137C mutation [5]. In addition, altered urinary excretion of AQP2 has been described in several human diseases of pathological AVP secretion. A decrease or increase in urinary AQP2 levels was shown to correspond with diminished or exaggerated levels of AVP, as seen in central diabetes insipidus or SIADH, respectively [3,4]. In normal individuals, water loading reduces antidiuresis and, as expected, urine AQP2 levels [3,15]. In studies of water loading in normal adults, urine AQP2 excretion at 2 hours after an oral water load of 20 mL/kg was reduced to 10% of baseline [15]. In the absence of appropriate diuresis during water loading, NSIAD patients would be predicted to have persistently elevated urine AQP2 levels. This is demonstrated in the R137C V2R mutation patient who received 50% of a standard water load [12] and confirmed by the results of our patient, who received the standard water load, in which urine AQP2 levels did not suppress normally, falling to only 87-98% of baseline for up to 3.5 hours after water loading.

The patient's serum AVP levels merit discussion. AVP levels were undetectable (< 1 pg/ml) on initial presentation with hyponatremia during infancy, leading us to the identification of an activating V2R mutation [5]. Following normalization of the patient's hydration status with fluid restriction and oral urea supplementation, AVP levels were in the normal range (5.5 pg/ml). AVP levels were again in the normal range (3.1 pg/ml) 6 months following self-discontinuation of urea, associated with eunatremia and hypodipsia. Furthermore, during the water loading challenge, serum AVP levels decreased from a baseline of 2.6 pg/mL to 1.1 pg/mL, the approximate assay limit of detectability, by one hour, when the patient had developed hyponatremia and serum hypoosmolality. Water loading in a patient with R137C mutation also resulted in low but detectable serum AVP level [10]. Thus, despite the presence of a constitutively active V2R, these results indicate appropriate regulation of AVP secretion in NSAID. Possible explanations for the low but detectable AVP levels following water loading in NSIAD include contamination with platelet-bound AVP [19] and/or a slight vasovagal stimulus that may have exerted a non-osmotic effect that resulted in an incomplete suppression of AVP secretion.

It is noteworthy that this patient demonstrated an intact thirst mechanism and relative hypodipsia, allowing him to maintain serum sodium in the 131-139 mmol/L range under *ad lib *conditions. His relative basal eunatremia is likely a consequence of a diet that is no longer exclusively liquid and is in marked contrast to the severe hyponatremia observed at initial presentation, when the infant was exclusively formula-fed. From a dietary standpoint, our results concur with a previous report in which a child with NSIAD, diagnosed retrospectively, remained eunatremic after transition to solid food and discontinuation of sodium supplementation [7,8,11].

An individual with NSIAD may escape detection during infancy if the activating V2R mutation is mild and/or if overhydration sufficient to induce hyponatremia does not occur. A report by Decaux *et al*. of a large pedigree of NSIAD patients caused by the R137C mutation in V2R highlights the marked variability in clinical presentation of this disorder [9]. One affected hemizygous male was apparently asymptomatic throughout life, and was discovered only after being administered a water loading test. Similarly, two other hemizygous adult males were discovered only when their 8 week old nephew was diagnosed with the disease and genetic testing was performed on the family [8]. One of the adult males was completely asymptomatic throughout his life and the other apparently has had generalized tonic-clonic seizures early in life of unknown etiology. In addition, several heterozygous females demonstrated inappropriate antidiuresis during water loading [8,10,12]. Thus, NSIAD may be more prevalent in the general population than would have otherwise been predicted based on the relatively small number of patients diagnosed in infancy.

In conclusion, this is the first report that urinary AQP2 levels in a patient with NSIAD caused by the R137L V2R mutation are elevated at baseline and do not suppress appropriately in response to water loading. This study demonstrates that urinary AQP2 can be a useful marker of V2R activity in NSIAD. Thus, increased urinary AQP2 excretion, in the setting of euvolemic hyponatremia and low or undetectable levels of serum AVP, suggests the possibility of NSIAD. This diagnosis should be confirmed by sequencing of *AVPR2*.

## Consent

Written informed consent was obtained from the patient for publication of this case report and any accompanying images. A copy of the written consent is available for review by the Editor-in-Chief of this journal.

## Competing interests

The authors declare that they have no competing interests.

## Authors' contributions

CCC and SMR designed the study, performed the analysis, and drafted the manuscript. MAC performed the western blot analysis. SAR and SEG participated in the design of the study. All authors read and approved the final manuscript.
